# Anti-inflammatory mechanism of Apolipoprotein A-I

**DOI:** 10.3389/fimmu.2024.1417270

**Published:** 2024-07-08

**Authors:** Xia Tao, Ran Tao, Kaiyang Wang, Lidong Wu

**Affiliations:** Department of Emergency, The Second Affiliated Hospital, Jiangxi Medical College, Nanchang University, Jiangxi, China

**Keywords:** Apolipoprotein A-I, macrophage, dendritic cell, neutrophil, T lymphocyte, anti-inflammatory

## Abstract

Apolipoprotein A-I(ApoA-I) is a member of blood apolipoproteins, it is the main component of High density lipoprotein(HDL). ApoA-I undergoes a series of complex processes from its generation to its composition as spherical HDL. It not only has a cholesterol reversal transport function, but also has a function in modulating the inflammatory response. ApoA-I exerts its anti-inflammatory effects mainly by regulating the functions of immune cells, such as monocytes/macrophages, dendritic cells, neutrophils, and T lymphocytes. It also modulates the function of vascular endothelial cells and adipocytes. Additionally, ApoA-I directly exerts anti-inflammatory effects against pathogenic microorganisms or their products. Intensive research on ApoA-I will hopefully lead to better diagnosis and treatment of inflammatory diseases.

## 1 Introduction

ApoA-I is a member of blood apolipoproteins, which constitutes a major component of HDL (1). In addition to participating in cholesterol reversal, ApoA-I also has powerful anti-inflammatory functions (2). It is considered an anti-inflammatory protein, with levels reduced by at least 25% during acute inflammation (3). ApoA-I attenuates the inflammatory response through inhibit the production of TNF-a and IL-1 in rheumatoid arthritis, Crohn’s disease and other immune diseases (4). A negative correlation between ApoA-I levels and the severity of pancreatitis has also been found in pancreatitis (5–7). Furthermore, our previous study revealed a negative correlation between ApoA-I levels and disease severity in hypertriglyceridemic pancreatitis (8). In cases of sepsis, there is a negative correlation between ApoA-I and the severity of the condition (9). Additionally, the administration of ApoA-I mimetic peptide has been shown to improve survival rates in septic rats (10). The anti-inflammatory function of ApoA-I also plays an important role in the inhibition of atherosclerosis and anti-tumor growth (11). A negative correlation between reduced ApoA-I and disease severity was also found in COVID-19 infections (12, 13). However, the exact anti-inflammatory mechanism of ApoA-I is not well understood. In this article, we review the production, assembly and possible anti-inflammatory mechanism of ApoA-I.

## 2 Production and assembly

**Figure 1:**
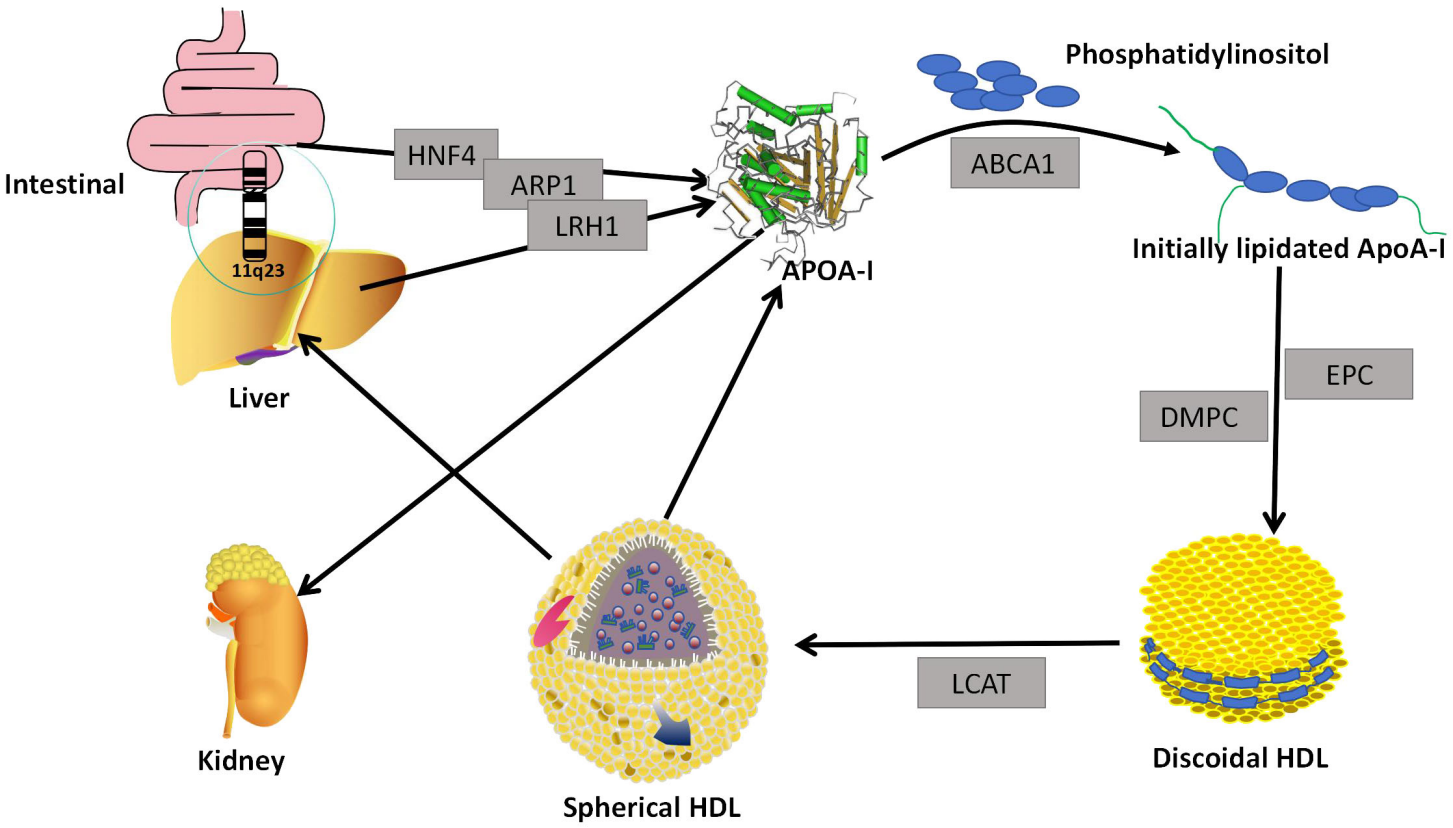
Generation and assembly. The ApoA-I gene located in the liver and small intestine secretes lipid-free ApoA-I under the regulation of HNF4, LRH1, and ARP1. Lipid-free ApoA-I binds to phosphatidylinositol to form Initially lipidated ApoA-I. Initially lipidated ApoA- I forms discoidal HDL in the presence of EPC and DMPC, and discoidal HDL forms globular HDL in the presence of LCAT. When globular HDL is reconstituted, lipid-free ApoA-I is formed. Most of the lipidated ApoA-I is metabolized in the liver, and the lipid-free ApoA-I is eliminated by the kidneys. HNF4, Hepatocyte Nuclear Factor4; ARP1, ApoA-I Regulatory Protein 1; LRH1, Liver Receptor Homologue 1; ABCA1, ATP-binding Cassette transport A1; DMPc, Dimyristoyl Phosphatidylcholine; EPC, egg phosphatidylcholine; LCAT, lecithin-cholesterol acyltransferase.

The human ApoA-I gene is located in the 11q23 region of human chromosome 11,and is thought to be of the same genetic origin as Apolipoprotein A-II, Apolipoprotein A-IV, Apolipoprotein C-I, Apolipoprotein C-III, and Apolipoprotein E (14). The liver and intestine are the main sites of ApoA-I production in human tissues, but small amounts of ApoA-mRNA expression have been demonstrated in other organs, such as the pancreas and heart (15). The regulation of human ApoA-I gene expression is very complex and is controlled at multiple levels. Hepatocyte Nuclear Factor 4, Liver Receptor Homologue 1 and ApoA-I Regulatory Protein 1 are thought to be the major regulators of ApoA-1 initiation and repression (16). Translated ApoA-I is pruned intracellularly and then secreted as a lipid-poor protein or lipid-free protein (**Figure 1**). ApoA-I gene mutations have been demonstrated to be associated with the development of numerous diseases, including those observed in diabetic patients. In particular, polymorphisms at the -75 bp locus of the ApoA-I gene have been linked to an increased risk of myocardial infarction in these patients (17). Individuals with the CC genotype of SNP rs5069 are more susceptible to oxidative imbalance and are at a higher risk of developing pancreatitis (18).

The primary structure of ApoA-I consists of 4 Tryptophan (Trp), 21 Lysine (Lys), 5Histidine (His), 16 Arginase (Arg), 16 Aspartic acid (Asp), 10 Threonine (Thr), 15 Serine (Ser), 27 Glutamic acid (Glu), 10 Proline(Pro), 10 Glycine (Gly), 19 Alanine (Ala), 13 Valine (Val), 3 Methionine (Met), 37 Leucine (Leu), 7 Tyrosine (Tyr), and 6 Phenylalanine (Phe), a total of 243 amino acid residues to form a single-chain peptide, which molecular weight is 28 KDa (19). This single-chain polypeptide may contain a plurality of 11 and 22 repeating amino acid residues, and these residues are typically separated by Pro residues. Among them, the 22 repeating amino acid residues can form the α-helix of ApoA-I, and the secondary structure of each ApoA-I contains about 8-9 tandem alpha helices (20). These α-helix play an important role in the biological function of ApoA-I (21–23). ApoA-I binds to the first extracellular domains(ECD1) of ATP-binding Cassette transport A1 (ABCA1), resulting in the transfer of cholesterol and cytosolic phospholipids from the cell membrane to ApoA-I, which then forms an initially lipidated ApoA-I (24). Initially lipidated ApoA-I can continue to form discoidal HDL in the presence of Dimyristoyl Phosphatidylcholine and egg phosphatidylcholine or palmitoyloleyl phosphatidylcholine (25). Studies of discoidal HDL mainly come from vitro models, as it is short-lived and difficult to isolate in plasma (26). Initially, it was believed that the binding of the α-helix to phospholipids in discoidal HDL was in the form of a spiked hedge fence. The α-helix consists of 22 repeating amino acids centered on a repeating proline residue crossing the edges of a bilayer parallel to the acyl chain (27). However, with research progressed, the ‘two-band’ model of discoidal HDL was considered the best possible model of α-helix binding to phospholipids. In this model, two cyclic ApoA-I molecules wrap a phospholipid bilayer in an antiparallel orientation. Discoidal HDL convers to spherical HDL by lecithin-cholesterol acyltransferase (LCAT) (28) (**Figure 1**). The globular HDLsubpopulation can be classified according to size and density into five major subpopulations: HDL3c, HDL3b, HDL3a, HDL2a, and HDL2b. Each subpopulation is distinguished by its unique molecular composition and biological function (29). ApoA-I is floating in the phospholipid molecules and serves to regulate HDL diameter in globular HDL (30).

ApoA-I constitutes the primary constituent of HDL, representing approximately 70%,ApoA-II accounts for 15-20%, while the remaining proteins are amphiphilic (1). ApoA-I not only determines the size and shape of HDL but is also a functional protein, such as determining the composition of HDL soluble lipids, transporting cholesterol from peripheral cells, activating LCAT activity to convert circulating cholesterol to cholesteryl esters, and delivering cholesteryl esters to the liver or to steroidogenic tissues via cell surface receptors (2). Most of ApoA-I is found in the blood associated with lipoproteins, with only about 5-10% present in a non-lipoprotein-associated state. This non-lipidated ApoA-I can be obtained from HDL remodeling or triglyceride-rich lipoproteins, or secreted directly by the liver or intestines (31). ApoA-I in plasma is about 100-150 mg/dl,and its half-life is about 4 days (32, 33). Most lipidated ApoA-I is metabolized in the liver (34), Non-lipidated ApoA-I can pass through glomerular filtration and be degraded, excreted in urine, or partially reabsorbed (35) (**Figure 1**).

## 3 Possible anti-inflammatory mechanism of ApoA-I

### 3.1 Regulation of immune cells

The inflammatory response is primarily determined on the function and activity of immune cells (36). Monocytes are an important component of the defense system of body, which play a crucial role in the immune system, and can induce a specific immune response in lymphocytes through antigen presentation (37). Macrophages are terminally differentiated monocyte, which have a variety of biological functions in the inflammatory response, including phagocytosis of microorganisms, mediation and promotion of the inflammatory response, antigen processing and presentation, modulation of the immune response, direct killing of target cells, adjuvant or inhibitory production of antibodies by B-lymphocytes, and production of cytokines (38). Immature dendritic cells have a strong migratory ability, and mature dendritic cells can effectively activate the initial T-cells to initiate the immune response (39). In the inflammatory response, neutrophils have multiple biological functions, such as chemotaxis, phagocytosis, apoptosis, degranulation, activation, production of reactive oxygen species and extra-neutral trapping networks (40). T cells play a key role in regulating the immune response, and which have responsible for mediating of the immune effector mechanisms (41).

**Figure 2:**
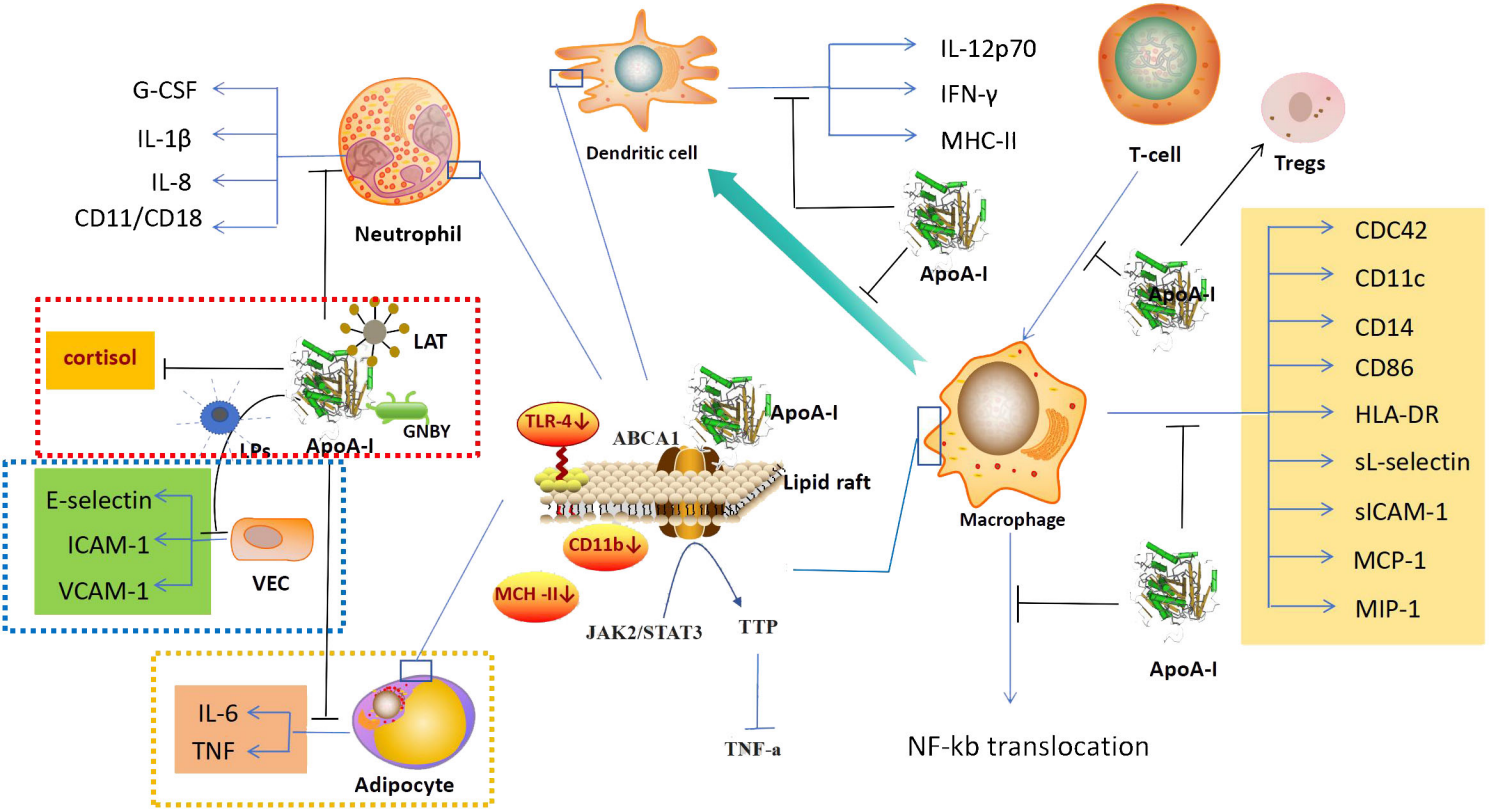
anti-inflammatory mechanism. In addition to its anti-inflammatory effects mainly through the regulation of immune cells,ApoA-I also exerts anti-inflammatory effects through the regulation of vascular endothelial cells (blue dashed area), the action on microorganisms and their products (red dashed area), and the regulation of adipocytes (yellow dashed area). In the regulation of immune cells, ApoA-I can inhibit the generation of immune cells, affect the expression of related factors, and inhibit the synergistic effect of immune cells. Its molecular mechanism is mainly to increase the efflux of cholesterol, regurate lipid raft-mediated signaling pathways and inhibit NF-kB nuclear translocation. ApoA-I, Apolipoprotein A-I; G-CSF, Granulocyte Colonystimulating Factor; IL-1β, interleukin 1β; IL-8, interleukin 8; LAT, Lipophosphatidic acid; LPs, lipopolysaccharide; GNBY, Gram-negative bacterium Yersinia; ICAM-1, Inter-cellular Adhesion molecule 1; VCAM-1, Vascular Cell Adhesion Molecule 1; VEC, Vascular endothelial cell; IL-6, interleukin 6; TNF, tumor necrosis factor; IL-12p70, interleukin 12p70; IFN-γ, Interferon γ; MHC-II, Major Histocompatibility Complex II; ABCA1, ATP-binding cassette transporter protein A1; TLR-4, Toll-like receptor; JAK2/STAT3, Janus kinase 2/Signal Transducer and Activator of Transcription 3; TTP, tristetraprolin; CDC42, Cell Division Cycle 42; HLA-DR, Human Leukoyte Antigen DR; sICAM-1, soluble Inter-cellular Adhesion molecule 1; MCP-1, Monocyte Chemotactic Protein 1; MIP-1, Macrophage Inflammatory Protein 1.

ApoA-I exerts a modulatory effect on the immune functions of monocytes, macrophages, dendritic cells, neutrophils, and T lymphocytes. It also suppresses inflammatory responses through multiple pathways (42, 43). First, ApoA-I can exert anti-inflammatory effects by regulating the production of immune cells. In a mouse model of acute myocardial infarction, ApoA-I has been observed to inhibit the expansion of monocytes and macrophages in the blood, spleen, and myocardium of mice (44). Upon leaving the bloodstream and entering tissues, monocytes can differentiate into Dendritic Cells(DCs),ApoA-I upregulates Prostaglandin E2 (PGE2) and Interleukin-10 (IL-10) in monocytes, and inhibits their differentiation to dendritic cells (45). In neutrophil generation, ApoA-I can reduce neutrophil production by decreasing the production of granulocyte colony stimulating factor (G-CSF) (46). Furthermore, ApoA-I has been demonstrated to reduce neutrophil counts in patients with acute myocardial infarction, resulting in less myocardial inflammatory injury (47). ApoA-I also has an effect on lymphocyte production, it deficiency leads to increase of CD45RA+, CD16+, and CD56+ lymphocytes in the blood (48). Transplantation of bone marrow from ApoA-I knockout mice into LDL receptor knockout mice resulted in a significant increase in lymphocytes (49). Second, ApoA-I can exert anti-inflammatory effects by regulating the expression and production of related factors of immune cells. The expression and production of related factors of immune cells are closely related to anti-inflammatory functions, such as spreading, recognition and chemotaxis. ApoA-I can directly inhibit the spreading and antigen-presenting ability of monocytes by down-regulating the expression of monocyte CDC42, CD11c, CD86, CD14, and HLA-DR (50, 51). Expression of vascular cell adhesion molecule-1 (VCAM-1), monocyte chemotactic protein 1 (MCP-1), and macrophage inflammatory protein 1 (MIP-1) was also inhibited by ApoA-I, which also significantly reduced the release of sL-selectin and soluble Inter-cellular Adhesion molecule 1(sICAM-1), and decreased monocyte chemotaxis, adhesion and activation function (52, 53). ApoA-I inhibits the synthesis of IL-8 by activated neutrophils, limiting their chemotaxis to local sites of inflammation (53). It was also found that ApoA-I reduces the expression of CD11b in neutrophils, leading to the decreased of adhesion, migration and spreading capacity of neutrophils (54). In polymorphonuclear leukocytes, ApoA-I was also found to have the ability to inhibit the expression of CD11/CD18, resulting in the decreased of adhesion of polymorphonuclear leukocyte(PMN) (55). Third, ApoA-I can exert anti-inflammatory effects by regulating the interaction of immune cells. In the inflammatory response, immune cells can coordinate and influence each other. As previously discussed, ApoA-I can affect the differentiation between monocytes and dendritic cells. ApoA-I also inhibits T-cell proliferation via DCs, and inhibits the reciprocal response between DCs and NK cells, resulting in decreased of IFN-γ and IL-12p70 (56). Fourth, It can exert anti-inflammatory effects by altering the expression of ApoA-I in immune cells. It has been found that ApoA-I is also expressed in macrophages, and the effect of its expression also affects macrophage function (57, 58). In monocytes, the expression of CD11b, CD11c, and CD29 was negatively correlated with ApoA-I levels in all monocyte subpopulations, and ApoA-I levels directly affected the anti-inflammatory activity of monocytes (59) (**Figure 2**).

Based on the available research, the regulation of the anti-inflammatory function of immune cells by ApoA-I may be achieved through the following molecular mechanisms: First, ApoA-I causes cholesterol efflux from immune cells to affect the expression of related factors. ApoA-I has a powerful cholesterol transporter function, it can modulate cellular biological functions by altering the cholesterol in cells, as previously demonstrated, the regulation of cholesterol in arterial vascular endothelial cells (60), and the recently discovered of regulation in pancreatic islet cells (61). ApoA-I has the same cholesterol-transporting effect in immune cells (62). Studies have shown that the decrease of CD11b and TLR-4 expression in monocytes by ApoA-I is due to the exocytosis of cholesterol from monocyte lipid rafts as a result of ApoA-I binding to ABCA1 in monocyte lipid rafts (52, 63), and the inhibition of macrophage antigen presentation and activation of T cells is also due to ApoA-I causing a decrease in the cholesterol content in macrophage lipid rafts (64). Second, ApoA-I affects the expression of related factors through lipid raft-mediated signaling pathways. After treating macrophages with ApoA-I, Yin Kai et al. found that the binding of ApoA-I to ABCA1 activated the Janus kinase 2/Signal Transducer and Activator of Transcription 3 (JAK2/STAT3),and upregulated the expression the tristetraprolin(TTP),which in turn promoted the degradation of TNF-a mRNA through AU-rich elements (AREs) in the 3-untranslated regions (3-UTR), leading to a reduction in the production of TNF-a and a decrease of inflammatory response (65). Third, suppression of gene activation in immune cells by inhibiting NF-kB nuclear translocation. NF-kB translocation plays a crucial role in intracellular signaling pathways and regulates various biological processes (66). In immune cells, stimulation by various factors can cause the transfer of the NF-kB subunit to the nucleus, activating numerous NF-kB-specific target genes, this activation leads to the initiation or enhancement of the immune response (67). ApoA-I inhibits NF-kB nuclear translocation in THP-1 differentiated monocytes, and the expression of VCAM-1 was inhibited and the release of sL-selectin and sICAM-1 were decreased. This reduced the chemotaxis, adhesion and activation functions of monocytes and inhibited the anti-inflammatory effects of monocytes (52, 68) (**Figure 2**). However, whether the initiating link of ApoA-I for NF-kB translocation inhibition is also due to ApoA-I affecting monocyte cholesterol is not known, and more studies are expected to further explore this.

### 3.2 Regulation of vascular endothelial cells

Vascular endothelial cells are a single layer of flat epithelium that covers the inner surface of blood vessels. During inflammation, vascular endothelial cells express adhesion molecules and interact with leukocyte surface adhesion molecules in the bloodstream. They also regulate leukocyte crossing of the vessel wall through signaling (69). In a study of a mouse model of type 2 diabetes revascularization, ApoA-I was found to reduce the inflammatory response of endothelial cells in the reconstructed blood vessels and promote vascular repair (70). ApoA-I can directly bind to LPs and influence its stimulatory effect on vascular endothelial cells. This results in the inhibition of E-selectin and intercellular adhesion molecule 1 (ICAM-1) expression in vascular endothelial cells, which leads to reduced adhesion of neutrophils to endothelial cells (56). *In vivo* experiments were conducted on New Zealand white rabbits infused intravenously with lipid-free ApoA-I, the results showed a reduction in the expression of VCAM-1 and ICAM-1, as well as a decrease in endothelial neutrophil infiltration (71) (**Figure 2**). However, the specific molecular mechanisms by which ApoA-I regulates vascular endothelial cell function are not well understood.

### 3.3 Action on microorganisms and their products

ApoA-I binds to a wide range of Gram-positive and Gram-negative bacteria, as well as to lipopolysaccharides and lipophosphatidic acids. In addition, ApoA-I has *in vitro* antimicrobial activity against Gram-negative bacteria (72). The C-terminal structural domain of ApoA-I serves as an effector site that provides bactericidal activity and contributes to complement mediated killing of the Gram-negative bacterium Yersinia enterocolitica in the small intestine (73) (**Figure 2**). In addition, ApoA-I was also found to possess *in vitro* antimicrobial properties against Gram-positive and Gram-negative bacteria in studies with tilapia, and ApoA-I inhibited inflammation and apoptosis and increased the likelihood of survival to bacterial infection (74), and its antimicrobial effect is not affected by temperature, even when the a-helix of ApoA-I is damaged by high temperature, ApoA-I still has the activity of killing bacteria directly (75). Therefore, the structure of ApoA-I may not be closely related to its antimicrobial activity.

Lipoteichoic acid (LTA) is an amphiphilic cationic glycolipid and a major cell wall component of Gram-positive bacteria. It has a structure similar to that of LPs from Gram-negative bacteria. LTA has been implicated as one of the major immunostimulatory components that may trigger systemic inflammatory response syndromes (76). ApoA-I significantly reduces L-929 cell mortality induced by LTA activated macrophages in a dose-dependent manner (77). LPs is one of the most potent stimulators of innate immune activation, and have important effects on human monocyte and macrophage (78). ApoA-I can neutralize the inflammatory effects of LPs through direct binding to LPs and can also regulate cortisol hormone production to play an anti-inflammatory role. In the sepsis model with ApoA-I knockout mice, it was found that ApoA-I knockout mice had a decreased ability to neutralize LPs compared to wild-type mice, and their serum cortisol hormone production was impaired, and the sepsis protection of the mice was reduced (79) (**Figure 2**).

### 3.4 Others

In adipocytes, ApoA-I inhibits the expression of IL-6 and TNF induced by palmitate, and it translocate TLR-4 to lipid rafts, thereby inhibiting inflammatory responses in adipocytes (80) (**Figure 2**).

## 4 The anti-inflammatory of 4F and CSL

Based on the robustness of ApoA-I and findings in the clinic, ApoA-I supplementation is considered a viable approach to treatment disease. Therefore, ApoA-I which can be used as a medicine has become the focus of attention. Such as ApoA-I mimetic peptides and ApoA-I based infusions.

Anantharamaiah et al. synthesized the first apoA-I mimetic peptide 18A in, 1985,which comprises 18 amino acids (81). Subsequently, the amino acid and α-helix structures of ApoA-I mimetic peptides have been modified to enhance their functionality (82, 83). To date, ApoA-I mimetic peptides in the pilot study phase are 4F,6F,FX-5A,ATI-5261 and ETC-642 (84). Among the many mimetic peptides, 4F has the most prominent anti-inflammatory effects. *In vitro*,4 F reduces IL-6 secretion in SARS-CoV-2 infected Vero-E6 and Calu3 cells (85), and it has an inhibitory effect on IL-4-induced selective activation of macrophages (43), 4F also inhibits Reactive Oxygen Species(ROS) production and ameliorates oxidative damage in endothelial cells (86). It has been shown in animal studies that ApoA-I mimetic peptides have the inflammation inhibition (87). In mice,4F inhibits the expression of interleukin-6(IL-6), interleukin-1β(IL-1β) and tumor necrosis factor-α (TNF-α) (88), it reduce macrophage infiltration in the liver (89). It also upregulates vascular endothelial growth factor protein expression to improve endothelial cell function in animal studies (90). An *in vivo* study in humans also showed that oral administration of apoA-I mimetic peptides 6F and 4F reduced plasma and intestinal tissue cytokines (TNF-a, IL-6) and chemokines (CX3CL1), and reduced systemic and intestinal inflammation in chronic treatment of HIV (91).

ApoA-I based infusions include HDL-VHDL infusion, purified ApoA-I infusion, ApoA-I_Milano_ infusion, CSL-111 and CSL-112 infusion,CER-001 infusion and modified ApoA-I (84). Of these,CSL-112 is considered the most viable infusion. CSL-112 consists of purified human ApoA-I and phosphatidylcholine, because it binds less phosphatidylcholine than CSL-111, it does not have the dose toxicity of CSL-111 (92). In previous studies, CSL-111 has been shown to have the effect of downregulating macrophages to reduce inflammation (93, 94). CSL112 also has shown anti-inflammatory effects ex vivo studies, including markers of monocyte chemotactic factor-1 and proinflammatory cytokines interleukin-1β (95).

## 5 Discussion

ApoA-I goes through a series of complex processes from generation to assembly (96). Thus, both the expression of the ApoA-I gene and the changes in its α-helix structure are susceptible to interference by external environmental factors, and these changes may be associated with the development of the disease (23, 97). ApoA-I is the primary component of HDL, and the biological functions of HDL, including cholesterol regulation (98), inflammatory response modulation (99), and tumor growth modulation are believed to be closely related to ApoA-I (100), which is an effective performer of HDL’s biological functions (101). As described above, ApoA-I plays a pivotal role in the inhibition of the inflammatory response, and this is achieved through the inhibition of immune cell production and activity, the reduction of the recognition of immune cells by vascular endothelial cells, and the direct destructive effect on microorganisms and their products. Specific molecular regulatory mechanisms have focused on the study of immune cells. According to the literature review, most of the regulatory effects of ApoA-I on immune cell function are related to its regulation of cholesterol, in which the effect on lipid rafts is an important target for ApoA-I to regulate immune cells, but a clearer and more systematic mechanism is still not well described, and more and more in-depth studies are expected to be conducted in the future. The decrease of ApoA-I has been detected in many diseases (102–105), especially in those related to inflammation (4–13). However, the underlying cause of the decrease is not clear. Whether the reduction is due to a decrease in ApoA-I production or an increase in ApoA-I depletions is necessary to explore.

As previously described, several studies have shown that ApoA-I mimetic peptides have some anti-inflammatory effects. However, even with studies showing that ApoA-I mimetic peptide does not play a role in suppressing inflammation. In the aortic constriction rabbit model, ApoA-I mimetic infusions did not significantly improve echocardiographic parameters nor molecular markers of cardiac inflammation, oxidative stress and fibrosis (106). This includes previous trial in patients with coronary artery disease or at high risk for cardiovascular disease that showed conflicting results on the effect of oral and parenteral administration of 4F on HDL inflammation indices (107). The contradictory test results may be attributed to the fact that the functions of the simulated components are distinct (108, 109) and that the final evaluation criteria differ for each test. ApoA-I mimetic peptide is a synthetic substance that is similar to ApoA-I. However, its molecular structure differs from ApoA-I, which means that it cannot exert the same effect when it enters the body. Furthermore, the pharmacokinetics and pharmacokinetics of ApoA-I mimetic peptides *in vivo* require further comprehensive and in-depth investigation. Research on the anti-inflammatory effects of ApoA-I mimetic peptides have been focused on *in vitro* and *in vivo* studies, with few clinical trials in humans. The main factors limiting the development of these may be related to the contradictory results of ApoA-I mimetic peptide tests and concerns about their safety. The development of ApoA-I mimetic peptides that are closer to the molecular structure of ApoA-I and safer may require more difficult work. However, from the theoretical standpoint, the structure of CSL-112 is more closely aligned with ApoA-I mimetic peptide, which should result in a more robust anti-inflammatory effect and enhanced safety profile. But the majority studies of CSL-111/112 on immune cells come from atheromatous plaque formation, lack of extensively studied as the ApoA-I mimetic peptides in inflammation. And concerns have been raised about the efficacy of CSL-112 due to the findings of recent clinical studies, which indicated that patients with acute myocardial infarction treated with CSL-112 did not experience a significant reduction in major adverse cardiovascular events (110). We are looking forward to deeper study and bigger breakthroughs in the anti-inflammatory treatment of CSL-112.

## 6 Conclusions

The production and assembly of ApoA-I is influenced by multiple factors, and ensuring the structural integrity of ApoA-I is a prerequisite for its anti-inflammatory effects. ApoA-I can exert its anti-inflammatory effects through regulate immune cells, vascular endothelial cells, and direct interaction with microorganisms and their products. Several experiments have demonstrated that ApoA-I mimetic peptides and ApoA-I based infusions inhibit inflammatory responses. It is anticipated that safer and more efficacious ApoA-I drugs will emerge in the near future as research progresses.

## Author contributions

XT: Writing – original draft. TR: Writing – original draft. KW: Conceptualization, Writing – review & editing. LW: Writing – review & editing.
